# Autosomal dominant cerebellar ataxia type I: A review of the phenotypic and genotypic characteristics

**DOI:** 10.1186/1750-1172-6-33

**Published:** 2011-05-28

**Authors:** Nathaniel Robb Whaley, Shinsuke Fujioka, Zbigniew K Wszolek

**Affiliations:** 1 Tri State Mountain Neurology 105 Woodlawn Dr Johnson City, TN, USA, 37604; 2 Mayo Clinic Jacksonville Department of Neurology 4500 San Pablo Rd Jacksonville, FL, USA, 32224

## Abstract

Type I autosomal dominant cerebellar ataxia (ADCA) is a type of spinocerebellar ataxia (SCA) characterized by ataxia with other neurological signs, including oculomotor disturbances, cognitive deficits, pyramidal and extrapyramidal dysfunction, bulbar, spinal and peripheral nervous system involvement. The global prevalence of this disease is not known. The most common type I ADCA is SCA3 followed by SCA2, SCA1, and SCA8, in descending order. Founder effects no doubt contribute to the variable prevalence between populations. Onset is usually in adulthood but cases of presentation in childhood have been reported. Clinical features vary depending on the SCA subtype but by definition include ataxia associated with other neurological manifestations. The clinical spectrum ranges from pure cerebellar signs to constellations including spinal cord and peripheral nerve disease, cognitive impairment, cerebellar or supranuclear ophthalmologic signs, psychiatric problems, and seizures. Cerebellar ataxia can affect virtually any body part causing movement abnormalities. Gait, truncal, and limb ataxia are often the most obvious cerebellar findings though nystagmus, saccadic abnormalities, and dysarthria are usually associated. To date, 21 subtypes have been identified: SCA1-SCA4, SCA8, SCA10, SCA12-SCA14, SCA15/16, SCA17-SCA23, SCA25, SCA27, SCA28 and dentatorubral pallidoluysian atrophy (DRPLA). Type I ADCA can be further divided based on the proposed pathogenetic mechanism into 3 subclasses: subclass 1 includes type I ADCA caused by CAG repeat expansions such as SCA1-SCA3, SCA17, and DRPLA, subclass 2 includes trinucleotide repeat expansions that fall outside of the protein-coding regions of the disease gene including SCA8, SCA10 and SCA12. Subclass 3 contains disorders caused by specific gene deletions, missense mutation, and nonsense mutation and includes SCA13, SCA14, SCA15/16, SCA27 and SCA28. Diagnosis is based on clinical history, physical examination, genetic molecular testing, and exclusion of other diseases. Differential diagnosis is broad and includes secondary ataxias caused by drug or toxic effects, nutritional deficiencies, endocrinopathies, infections and post-infection states, structural abnormalities, paraneoplastic conditions and certain neurodegenerative disorders. Given the autosomal dominant pattern of inheritance, genetic counseling is essential and best performed in specialized genetic clinics. There are currently no known effective treatments to modify disease progression. Care is therefore supportive. Occupational and physical therapy for gait dysfunction and speech therapy for dysarthria is essential. Prognosis is variable depending on the type of ADCA and even among kindreds.

## Disease name/synonyms

Autosomal Dominant Cerebellar Ataxias, Spinocerebellar ataxias.

## Disease definition/Diagnostic criteria

The definition of spinal cerebellar ataxias (SCAs) despite significant progress in their understanding is still imprecise. They can be divided by the mode of inheritance to autosomal dominant, autosomal recessive, or sporadic conditions, Harding proposed a classification of autosomal dominant cerebellar ataxias (ADCA) into three categories, Type I, Type II and Type III. ADCA Type I comprises syndromes such as SCA1- SCA4, SCA8, SCA10, SCA12 - SCA23, SCA25, SCA27, SCA28 and DRPLA. ADCA Type II comprises syndromes associated with pigmentary maculopathies and includes SCA7. ADCA Type III comprises pure cerebellar syndromes and includes SCA5, SCA6, SCA11, SCA26, SCA29, SCA30 and SCA31 [1].

The ADCA Type I are the subject of this review. ADCA Type I contain at the time of this writing a group of 22 disorders. There are no fully validated diagnostic criteria for ADCA Type I. The diagnosis is based on clinical history, physical examination and genetic testing.

## Clinical and pathological classifications

Phenotypes of ADCA Type I are complex and include ataxia plus other neurological signs. The clinical spectrum ranges from just "pure" cerebellar signs to constellations including spinal cord syndromes, peripheral nerve disease, cognitive impairment, cerebellar or supranuclear ophthalmologic signs, psychiatric problems, and seizure disorders. The ataxia in ADCA Type I is characterized as disordered voluntary movement in (1) the rate of initiation and cessation called dyschronometria, (2) the amplitude known as dysmetria, (3) the coordination of single movements termed dyssynergia, (4) the speed of alternating movements called dysdiadochokinesia, and (5) the continuity resulting in action tremors [2]. Cerebellar ataxia can affect virtually any body part causing movement abnormalities. Gait, truncal, and limb ataxia are often the most evident cerebellar findings though nystagmus, saccadic abnormalities, and dysarthria are usually associated. Table 1 lists clinical signs commonly observed in ADCA Type I. Generally, the ADCA Type I manifest in adulthood; however, presentation in childhood may occur. This can be the result of the phenomenon of anticipation that results from trinucleotide repeat expansion mutations that may lengthen in subsequent generations particularly if transmitted paternally. The size of the repeat in those associated with trinucleotide repeat expansions may be inversely related to symptomatic age-related onset [2].

**Table 1:** Clinical signs in ADCA type 1

Clinical Signs (other than ataxia)	*ADCA type 1*
Slow Saccades	SCA1, SCA2, SCA3, and SCA7
Ophthalmoplegia	SCA1, SCA2 and SCA3
UMN signs	SCA1, SCA3, and SCA12 (sometimes SCA8)
Extrapyramidal	SCA3, and SCA12 (Parkinsonism) SCA3 (Dystonia) SCA2, and occasionally SCA1, SCA3, and SCA19(Myoclonus) SCA12, SCA16, SCA19
Cortical	SCA13, SCA21 SCA17 (Dementia, psychosis, and epilepsy)
Pontine Signs	SCA1, SCA2, and SCA3
Fasciculations	SCA3
Peripheral Neuropathy	SCA3, SCA4, SCA18

ADCA = autosomal dominant cerebellar ataxia; SCA = spinocerebellar ataxia; UMN = upper motor neuron

Pathologically the different combination of degeneration of the cerebellum, spinal tracts, peripheral nerve, cerebral cortex, basal ganglia, pontomedullary systems, optic nerve, and others is seen [3].

## Epidemiology

The prevalence of the SCAs as a whole is similar to Huntington disease and is estimated to be 2-3 per 100,000 people but may be as high as 5-7 in 100,000 in some populations [4-13], though the prevalence of ADCA Type I is unknown. The most common ADCA Type I is SCA3 followed by SCA2, SCA1, and SCA8 in descending order [7]. Founder effects doubtless contribute to the variable prevalence between populations.

## Pathogenesis

The pathogenesis of the ADCA Type I is not fully understood. Categories of ADCA Type I based on proposed pathogenesis have been suggested [14]. Accordingly, there are 3 major subclasses. The first and probably most common subclass includes SCA1, SCA2, SCA3, and SCA17; and DRPLA that are associated with trinucleotide CAG repeat expansions encoding large uninterrupted glutamine tracts. The prevailing explanation for the mechanism of neuronal injury observed in these syndromes is that the polyglutamine product is in some way toxic to the cell at the protein level. The details supporting this hypothesis are beyond the scope of this discussion but have been reviewed recently [3,15]. In brief, the toxic effect may be mediated by interference of protein aggregation and clearance, transcriptional dysregulation, alteration of the ubiquitin-proteasome system, and perturbance of calcium homeostasis leading to premature apoptosis [14]. The second pathogenic subclass includes those ADCA Type I such as SCA8, SCA10 and SCA12 related to trinucleotide repeat expansions that fall outside of the protein-coding region of the disease gene. Again, a toxic reaction is the proposed cause of neuronal damage though in this situation at the RNA level. A similar phenomenon is behind the pathogenic mechanism for the Fragile X-associated tremor ataxia syndrome. These RNA repeat sequences interfere with gene expression in neurons [14]. The last sub-class encompasses SCA13, SCA14, SCA15/16, SCA27 and SCA28 caused by specific gene deletions, missense mutations, and nonsense mutations leading to neurodegeneration [14].

Interestingly, despite the differences in the proposed mechanisms, the phenotypes of the syndromes in all three subclasses is remarkably indistinguishable on clinical grounds. This would seem to suggest that the disruption of a final common pathway leading to neurodegeneration can be mediated through a number of cellular pathways.

## Diagnosis

Definitive diagnosis rests on genetic analysis though the importance of the history of illness including a detailed family history and physical examination can not be overstated. Deriving a specific diagnosis based on genetic testing is beneficial primarily for individuals of childbearing age where genetic counseling would be required as there are no known cures for the ADCA Type I. Strategies for selecting the genetic test or tests most likely to result in diagnosis have been suggested and may prove beneficial in reducing costs as these tests are too expensive to send for indiscriminately and are often not reimbursed by insurance companies [2]. Though the classification of Harding may be somewhat outdated, it is practical one and allows the clinician at bedside to prioritize genetic testing. Importantly, treatable causes of cerebellar ataxia must be excluded and screening for these conditions should be part of the evaluation of any individual presenting with cerebellar signs and symptoms. Table 2 includes a differential diagnosis of cerebellar ataxia.

**Table 2:** Differential diagnosis of cerebellar ataxia other than ADCA

**Drug Effect**	Phenytoin, 5-fluorouracil, cytosine arabinoside, bismuth (Pepto-Bismol^®^), mercury-containing fungicides, and lithium
**Toxin**	Ethanol, methyl mercurial compounds, solvents containing toluene and metals such as lead, manganese, and tin
**Nutritional**	Vitamin E deficiency, thiamine deficiency (Wernike-Korsakoff syndrome), Gluten sensitivity (Celiac sprue)
**Endocrinopathy**	Hypothyroidism and hypopituitarism
**Infection**	HIV, varicella, Epstein-Barr, prion (Creutzfeldt-Jakob, Kuru etc.)
**Postinfection**	Guillain-Barre and Bickerstaff's encephalitis
**Structural or lesional**	Ischemic infarction, hemorrhage, neoplasm, demyelination, abscess etc.
**Neurodegeneration**	Multiple systems atrophy and recessively inherited cerebellar ataxias
**Paraneoplastic**	Small cell, breast, and ovarian cancer
**Other**	Idiopathic late onset cerebellar ataxia and ataxia with antiglutamate decarboxylase antibodies

ADCA = autosomal dominant cerebellar ataxia; HIV = human immunodeficiency virus

**Figure 1:**
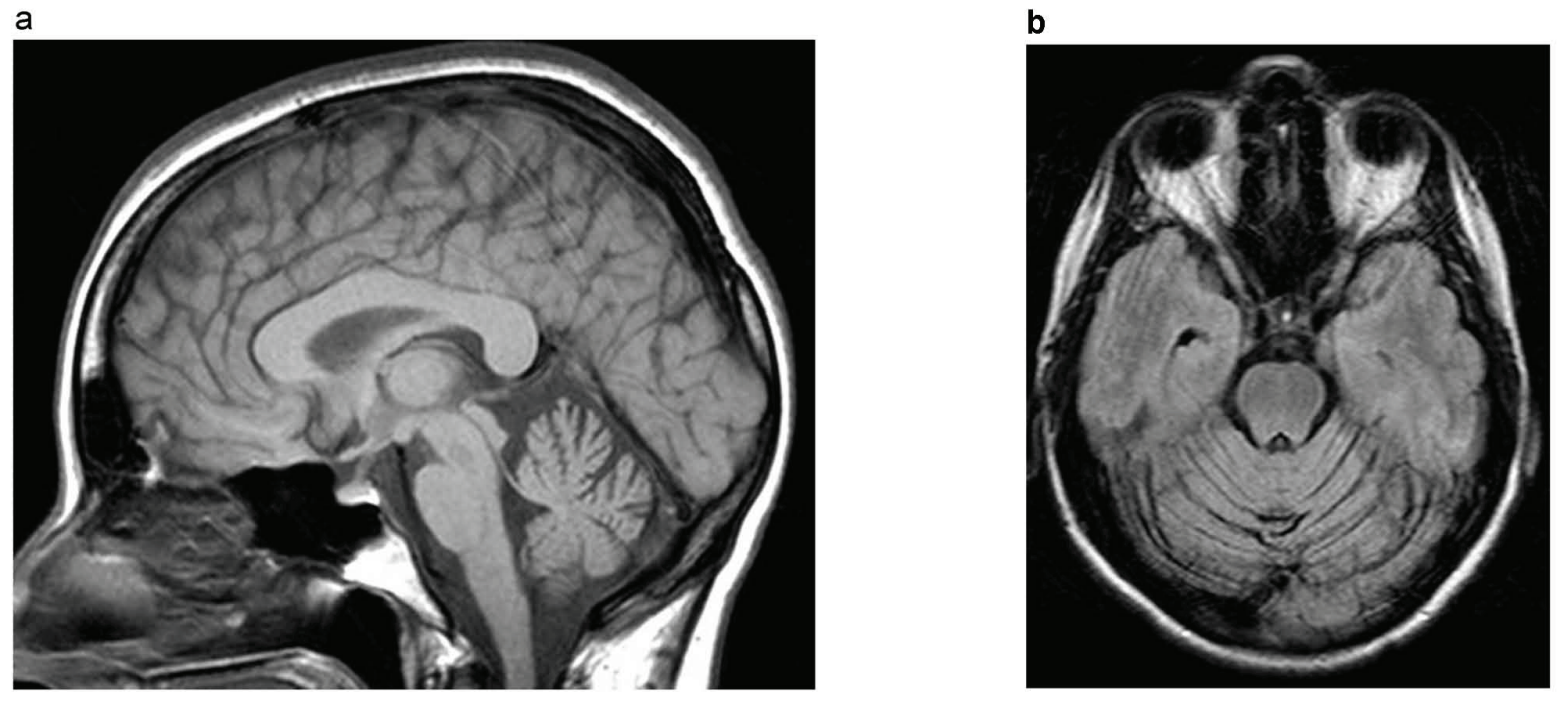
**The MR (T1) brain (A) sagittal, (B) axial in a 54 year old African-American male with clinically genetically confirmed SCA2 who presented with cerebellar ataxia, dysarthria, slowed saccades, and peripheral neuropathy demonstrates cerebellar and brainstem atrophy**.

Neuroimaging except for the purpose of excluding other causes of cerebellar ataxia is of little value to the clinician in distinguishing between the ADCA Type I. Magnetic resonance imaging (MRI) is superior to computed tomography (CT) for this purpose. Unfortunately, no pattern of atrophy on MRI is specific for a particular genotype of ADCA Type I [16]. Cerebellar atrophy is a typical finding with or without brainstem or cortical atrophy (Figure 1). Not surprisingly, areas of apparent neurodegeneration on MRI have been correlated with clinical signs referable to the areas of atrophy [17].

Pathological studies in individuals with ADCA Type I are scant. Studies have demonstrated variable neuronal loss and gliosis in regions of the rhombencephalon more than diencephalon and telencephalon and spinal cord [18]. Like neuroimaging, pathological findings are not specific and are insufficient in distinguishing between the ADCA Type I genotypes.

## Management

Supportive care remains the mainstay of management as there is no cure for any of the ADCA Type I at this time. Occupational and physical therapy for gait dysfunction and speech therapy for dysarthria is essential. The use of mechanical aids such as a cane, walker or wheelchair is beneficial in maintaining safety with ambulation and freedom of mobility for as long as possible. Other symptoms such as insomnia, diplopia, spasticity, and urinary urgency or frequency should be treated accordingly in order to improve quality of life [2]. Depression is a potentially treatable and relatively common presenting symptom particularly in SCA3 and should be evaluated for and treated aggressively when present [19]. Likewise, pain particularly related to dystonia can be severe and is an often overlooked component of the ADCA Type I that may be misdiagnosed and mistreated but successfully ameliorated by botulinum toxin therapy [20].

## Prognosis

These disorders are unrelentingly progressive and can shorten length of life [21-23]. However, the prognosis is variable between ADCA Type I and even among kindreds. The longitudinal studies have not been performed. Accurate prognostication for an individual patient is difficult to ascertain; however, the determination should begin with the assessment of the phenotype observed in the individual's family and consider the proband's age at presentation. A younger age of onset and longer trinucleotide repeat length generally portends a poorer prognosis.

## Clinical description of subforms

### Spinocerebellar Ataxia Type 1 (SCA1)

SCA1 typically presents in the 4^th ^decade (age range is 4 and 74 years) with dysarthria, hand writing difficulties, and limb ataxia [18,24]. Nystagmus and saccadic abnormalities are common. As the syndrome progresses so does the ataxia, but additional features may emerge and include proprioceptive loss, hypoactive reflexes, ophthalmoparesis, and mild optic neuropathy [25]. Initial presentation with blepharospasm, oromandibular dystonia, and retrocollis preceding ataxia has been reported [26]. Cognition is relatively spared early on; however, executive dysfunction and impaired verbal memory may develop in later stages [27]. In the end stages, usually 10 to 15 years following onset, bulbar dysfunction secondary to affection of lower medullary nuclei results in aspiration and death [28].

SCA1 was the first ADCA Type I to be genetically classified when Yakura et al. linked this form of ataxia with the HLA complex and chromosome 6p in 1974 [29]. In 1987, the *SCA1 *gene was mapped to a region 15cM distal to the HLA complex [30]. By 1993, the *SCA1 *critical region was mapped to a <2cM segment on 6p [31]. Later that year the gene was cloned, and the mutation was determined to be an unstable CAG trinucleotide repeat within the gene's coding region resulting in an expanded tract of glutamine amino-amino acids [32]. *SCA1*'s product, ataxin-1, results in variable CAG repeats ranging from 6 to 44 in the general population, and those with longer repeats (>20) typically have CAT triplet interruptions within the CAG tract [33]. In contrast, affected alleles have repeat expansions over 39 devoid of these CAT triplet interruptions. The pathogenesis of polyglutamine related syndromes is discussed above. Diagnosis is confirmed by DNA analysis demonstrating over 39 uninterrupted CAG repeat expansions in the *SCA1 *gene region on chromosome 6p.

### Spinocerebellar Ataxia type 2 (SCA2)

SCA2 presents in the third or fourth decade (average age 30 years; age range is from 2 to 65 years) with truncal ataxia, dysarthria, slowed saccades and less commonly ophthalmoparesis and chorea [34]. Parkinsonism is also a less common but well documented manifestation of SCA2 [35]. Despite this, there is no distinct clinical feature that reliably distinguishes this from SCA1. Recent studies suggest that mitochondrial gene polymorphism and a polyQ repeat variation in the CACNA1A calcium channel may play a role in the clinical variability observed in individuals with SCA2 [36,37].

SCA2 patients were found worldwide, and is among the three most frequent types of ADCA Type I, together with SCA3 [38]. In 1993, Gispert et al. located the locus for SCA2 in a Cuban kindred that mapped to chromosome 12 [39,40]. Since then, the gene responsible for SCA2, ataxin 2, has been identified as a novel cytoplasmic protein; its function is yet unknown [41-43]. The normal CAG repeat length is 15-24. Repeats 35 and longer are associated with the clinical syndrome. Like SCA1, anticipation and an inverse relationship between age at onset and repeat length is observed in kindreds. Likewise, CAG repeats are uninterrupted in affected individuals.

### Spinocerebellar Ataxia type 3 (SCA3)

Also known as Machado-Joseph disease, this is likely the most common SCA in most populations genetically characterized to date and can be classified into three clinical phenotypes [44]. Type 1 is associated with ataxia, ophthalmoparesis, pyramidal signs such as spasticity and hyperreflexia, and extrapyramidal signs including dystonia and other movement disorders presenting in adolescence. Individuals with Type 2 present in middle adulthood with ataxia, spasticity, and dystonia. Type 3 occurs after the age of 40 and includes ophthalmoparesis and anterior horn cell disease i.e. fasciculations, atrophy, and weakness. Parkinsonism can also be a feature of SCA3 [44]. A likely overlooked but common feature is impairment of temperature sensation involving the entire body [45].

The mutation is a CAG repeat expansion in chromosome 14q24.3-q32.1 [46]. The normal repeat length is 13-41 whereas repeat lengths causing SCA3 are greater than 56 [47-49]. SCA3 gene-product, ataxin 3 is a deubiqutinating enzyme that edits topologically complex chains [50] and found in the cytoplasm of many types of cells outside of the CNS. Ataxin 3 binds Lys48-linked ubiquitin chains, and then cleaves Lys63 linkages and possibly other non-Lys48 linkages. This activity would help ensure efficient proteasomal degradation of ubiquitinated substrates [50].

### Spinocerebellar Ataxia type 4 (SCA4)

SCA4 is commonly referred to as hereditary ataxia with sensory neuronopathy and was first described by Biemond in 1954 [51]. SCA4 is relatively rare syndrome, the kindreds were found in USA (Scandinavian origin and residing in Utah and Wyoming), Germany and Japan. This syndrome typically starts in middle age adults and presents with cerebellar ataxia, pyramidal signs, and peripheral sensory loss [52]. SCA4 has been linked to chromosome 16q22.1 in kindreds from Utah and Germany [53,54]. The mutation is yet unknown but does not appear to be a trinucleotide repeat disorder though anticipation has been suggested in both kindreds. Interestingly, a Japanese kindred with a pure cerebellar ataxia phenotype without sensory loss and with an older age of symptomatic onset has been linked to the same region on chromosome 16 and is strongly associated with a C to T substitution in the puratrophin 1 gene [55,56]. This syndrome known as 16q-ADCA appears to be clinically distinct from SCA4.

### Spinocerebellar Ataxia type 8 (SCA8)

SCA8 presents in adulthood with cerebellar ataxia plus cognitive dysfunction in as many as 71% and pyramidal and sensory signs in approximately a 3^rd ^of affected individuals [57]. The age of onset is between 0 to 73 years (mean age of onset is 38.3 years). The kindred has a worldwide distribution, especially in Europe. A dysexecutive syndrome has been a reported feature as has a significant occurrence of psychiatric diagnoses [57,58].

First described in 1999, SCA8 is now known to be associated with a trinucleotide repeat on 13q21 that produces a polyglutamine expansion, ataxin 8 [59]. Ataxin 8 is transcribed as a part of an untranslated that serves as a gene regulator [60]. The pathogenesis of SCA8 is thought to result from RNA-mediated neurotoxicity [3]. It is also distinguishable because it is associated with a CTG repeat expansion similar to myotonic dystrophy. The syndrome has been associated with 107 to 127 CTG repeat expansions. This repeat is bidirectionally transcribed resulting in both a noncoding CUG transcript and a CAG transcript resulting in a pure polyglutamine protein that forms inclusions in mice and humans [61]. Expansions on 13q21 in 0.7% of nonataxic controls have lead to controversy regarding a true association between the syndrome and the expansion at this site [62]. Recent reports appear to confirm an association [57].

### Spinocerebellar Ataxia type 10 (SCA10)

SCA10 is characterized by slowly progressive cerebellar syndrome and epilepsy, sometimes mild pyramidal signs, peripheral neuropathy and neuropsychological disturbances are also present. The most common type of epilepsy is generalized motor seizures, but partial motor or partial complex seizures can occur [63]. The age of onset ranges from 18 to 45 years (mean age is 32.2 years) [63,64]. Many kindreds has been found in Mexican and Brazilian populations, SCA10 is second common inherited ataxia in these two countries. Recently, Argentinian family [65] and additional Latin American families [66] have been reported. SCA10 is caused by an ATTCT pentanucleotide repeat expansion in intron 9 of the *ATXN10 *gene. The expanded alleles range from 800 to 4500, whereas the normal alleles from 10 to 29 [67]. The pathogenesis has not been elucidated, but processing of RNA may be involved [68].

### Spinocerebellar Ataxia type 12 (SCA12)

The hallmark of SCA12 is the presence of action tremor associated with a relatively mild cerebellar ataxia [69]. Associated pyramidal and extrapyramidal signs and dementia have been reported [70]. The age of symptomatic onset ranges from 8 to 55 with most individuals presenting in the fourth decade [70].

SCA12 was first identified in a German-American kindred and more recently has been described in India where a common founder has been identified [70,71]. Though SCA12 is very rare, except for a single ethnic group in India, two Italian families have been identified recently [72]. Along with SCA8, the pathogenesis of SCA12 seems to be related to a toxic effect at the RNA level. SCA12 is associated with a CAG expansion at the 5' end of the gene encoding PPP2R2B of chromosome 5q31-5q32 [71]. PPP2R2B encodes a brain-specific regulatory subunit of the protein phosphatase 2 [73]. The number of repeat expansions associated with this syndrome is between 55 and 78 triplets [70].

### Spinocerebellar Ataxia type 13 (SCA13)

Originally described in a French kindred, later in a Filipino family and two additional European families, the salient feature of SCA13 is onset in childhood marked by delayed motor and cognitive development followed by mild progression of cerebellar ataxia [74-76]. While primarily a cerebellar syndrome, dysphagia, urinary urgency, and bradykinesia have been described in affected individuals older than 50.

SCA13 has been mapped to chromosome 19q13.3-q13.4 and is now known to be associated with two missense mutations in the *KCNC3 *gene in this region [77]. This gene encodes a voltage-gated potassium channel not previously identified with neurodegeneration.

### Spinocerebellar Ataxia type 14 (SCA14)

SCA14 presents in early adulthood with wide range of symptomatic disease onset from 10 to 70 years (mean 33.9 years). The phenotype of SCA14 is mild and encompasses slowly progressive ataxia, dysarthria and nystagmus. In addition to the cerebellar signs, hyperreflexia and decreased vibration sense are frequently observed. Some patients have a cognitive impairment, parkinsonism characterized by rigidity [78] as well as focal dystonia [79], axial myoclonus [80,81], facial myokymia [82], choreic movement of hands [82] and epilepsy [83].

SCA14 was initially identified in a 4-generation Japanese kindred [80]. At the present time there are published reports of more than twenty families from Europe, the USA, and Australia [78,82,84,85]. SCA14 is caused by missense mutations in the PRKCG gene encoding protein kinase Cγ (PKCγ) [84]. PKC is a family of serine-and threonine kinases with PKCγ being one of the members. PKCγ is expressed abundantly in the neurons especially in Purkinje cells [86] and is thought to play important roles in signal transduction, cell differentiation, and synaptic transmission [82].

### Spinocerebellar Ataxia type 16 (SCA16) and type 15 (SCA15)

SCA16 was described in a single family from Japan in 2001. Phenotype was reported as a "pure" cerebellar ataxia [87]. Since then, cognitive dysfunction has been noted in affected individuals [88]. The age of onset is from 20 to 66 years with mean of 39.6 years. In this Japanese family SCA16 has been initially linked to chromosome 3p26.2-pter and a single nucleotide substitution (4,256C→T) on contactin 4 (*CNTN4*) gene was proposed in 2006 [88]. However, further studies performed in 2008 revealed that this substitution represents a rare polymorphism [89].

Earlier in 2001, SCA15 was identified in Australian family with "pure" cerebellar ataxia, thus falling into the broad Harding classifications as ADCA Type III [90]. In 2004, additional two SCA15 families were identified in Japan with affected individuals having cerebellar ataxia and some also exhibiting postural and action tremor [91]. In the same year a longitudinal clinical observations of the Australian kindred were published reporting that all affected family members had developed a cognitive impairments [92]. In 2007, Leemput et al. detected the deletion on inositol 1,4,5-triphosphate receptor 1 (*ITPR1) *gene and sulfatase-modifying factor 1 (*SUMF1) *gene in three SCA15 families including the above mentioned Australian kindred and in two newly identified British families [93]. In 2008, Iwaki et al reported that in their original Japanese family described in 2001 the genetic defect represents the same deletion on *ITPR1 *gene as seen in Australian and British kindreds further strengthening the argument that both SCA16 and SCA15 are indeed due to *ITPR1 *gene mutations [89,94]. This year a British family with ataxia unrelated to previously reported British kindreds was described [95]. The protein analysis demonstrated the *ITPR1 *deletion [95]. The study by Iwaki et al reveled that previously implicated *SUMF1 *gene is not responsibly for clinical phenotype in SCA15/16 [89]. Therefore, SCA15/SCA16 are produced by the same genetic dysfunction and should be categorized on the base of predominant clinical phenotype as part of ADCA Type I.

### Spinocerebellar Ataxia type 17 (SCA17)

The phenotype of SCA17 is particularly variable and can be associated with dementia, psychiatric disorders, parkinsonism, dystonia, chorea, spasticity, and epilepsy [16]. Clinical features overlap with many neurodegenerative syndromes and Huntington disease specifically.

First recognized in 2001, this syndrome has since been mapped to chromosome 6 and is secondary to a CAG repeat expansion in the TATA box binding protein gene (*TBP*) [96]. *TBP *encodes for a general transcription initiation factor. SCA17 mutation has been reported in families of Japanese, German, French, Chinese, Korean, Italian, Mexican, Taiwanese, and Indian descent but its frequency is rather low compared to SCA1 - SCA3 [97]. In a recent report, areas of atrophy on MRI in individuals with SCA17 were associated with clinical signs referable to those areas [16]. In addition, this study revealed low Mini-Mental State Examination scores correlated with atrophy of the nucleus accumbens.

### Spinocerebellar Ataxia type 18 (SCA18)

Also known as autosomal dominant sensory/motor neuropathy with ataxia, SCA18 presents initially with an axonal sensory neuropathy with cerebellar ataxia and motor neuron dysfunction developing later [98]. SCA18 presents in the second and third decades of life with symptomatic disease onset ranging from 13 to 27 years [98]. Initially this syndrome was described in an American-Irish family and has been linked to chromosome 7q22-q23 [99]. The responsible gene mutation has not been identified. Both SCA3 and SCA4 are also associated with a peripheral neuropathy.

### Spinocerebellar Ataxia type 19 (SCA19) and Spinocerebellar Ataxia type 22 (SCA22)

SCA19 is a syndrome identified in a Dutch kindred in 2001. SCA19 presents in the third decade of life with symptomatic disease onset ranging from 10 to 46 years. The phenotype is characterized by mild cerebellar ataxia, cognitive impairment, low scores on the Wisconsin Card Sorting Test, myoclonus, and postural tremor [100]. Linkage to chromosome 1p21-q21 has been proposed; however, the gene mutation has not been identified [101].

SCA22 was reported in a Chinese pedigree in 2003. SCA22 symptomatic disease onset overlaps significantly with symptomatic disease onset of SCA19 but with more narrow range of 35 to 46 years. Clinical features usually include only cerebellar signs. Occasionally hyporeflexia is present. Linkage to chromosome 1p21-q23 has been made [102], but Schelhaas et al. hypothesized that SCA19 and SCA22 share the same genetic locus [103].

### Spinocerebellar Ataxia type 20 (SCA20)

SCA20 is a syndrome identified in a pedigree of Anglo-Celtic origin in 2004 [104]. A cerebellar dysarthria is typically the initial manifestation. However, most of the affected persons also exhibit palatal tremor and spasmodic dysphonia. Head CT shows the dentate calcifications. The age of symptomatic disease onset ranges from 19 to 64 years (mean age; 46.5years). SCA20 has been linked to chromosome 11q12.2-11q12.3 [105] overlapping with locus for SCA5, though clinical features differ between SCA5 and SCA20. SCA5 belongs to ADCA Type III and represents a "pure" ataxia syndrome with on average earlier age of symptomatic disease onset ranging from 14 to 50 years) [106,107]. Since the causative gene is unknown, it may well be that after discovery of such gene SCA20 and SCA5 may be indeed genetically proven to be the same disorder as it has already occurred with SCA16 and SCA15 (discussed above).

### Spinocerebellar Ataxia type 21 (SCA21)

Identified only in a French kindred, SCA21 causes slowly progressive cerebellar ataxia, mild cognitive impairment, postural and/or resting tremor, bradykinesia, and rigidity [108,109]. Age of onset is 17.4 years and is relatively earlier than for most ADCA Type I SCAs. The parkinsonism was not responsive to L-dopa [109]. MRI revealed cerebellar and brainstem atrophy. SCA21 maps to chromosome 7p21.3-p15.1 [109]; however, the gene and gene mutation has not been identified. Individuals in successive generations tend to have earlier ages of onset [109].

### Spinocerebellar Ataxia type 23 (SCA23)

SCA23 has been identified only in one Dutch family. SCA23 is characterized by gait ataxia, dysarthria, slowed saccades, ocular dysmetria, Babinski signs and hyperreflexia [110]. Individuals with SCA23 have an age of onset from 43 and 56 years. SCA23 maps to chromosome region 20p13-12.3 [110]. The clinical features, head MRI, and neuropathological findings are indistinguishable from other SCA subtypes.

### Spinocerebellar Ataxia type 25 (SCA25)

Identified in a French kindred, SCA25 is characterized by cerebellar ataxia and prominent sensory neuropathy [111]. The clinical features vary widely from sensory neuropathy with little cerebellar ataxia to cerebellar ataxia with little sensory neuropathy. Some patients exhibit gastrointestinal dysfunction such as vomiting and abdominal pain as initial symptom. Digestion problems can be persistent. Scoliosis and urinary problems (nycturia or urinary urgency). Head MRI shows severe global cerebellar atrophy like in SCA5 and SCA6. The age of onset ranges from 1 to 39 years [111]. SCA25 maps to chromosome 2p15-p21 [111]. Repeat expansion detection failed to identify CAG repeat expansion.

### Spinocerebellar Ataxia type 27 (SCA27)

SCA27 was described in a Dutch family with early onset tremor, dyskinesia, and slowly progressive cerebellar ataxia associated with a mutation in the fibroblast growth factor 14 (*FGF14*) gene on chromosome 13q34 [112]. The mutation in this family was missense mutation; however, since then a frameshift mutation in the *FGF14 *gene has been described in an individual with a familial ataxia [113] and the mechanism of neurodegeneration resulting from this mutation is unknown.

### Spinocerebellar Ataxia type 28 (SCA28)

SCA28 is characterized by juvenile onset slowly progressive cerebellar ataxia due to Purkinje cell degeneration. Some persons show cognitive impairment [114]. And in more advanced stages of the syndrome, ophthalmoparesis, slowed saccades, ptosis and pyramidal signs were reported [115]. The mean age of symptom onset was 19.5 years in the original kindred. Additional kindreds were found only in Europe [114,115]. SCA28 accounts for approximately 1.5% of all European ADCA patients [116]. The candidate disease locus was identified on chromosome 18p11.22-q11.2 [115]. Recently missense mutation in the ATPase family gene 3-like 2 (*AFG3L2*) has been discovered. AFG3L2 is a component of the conserved matrix ATPase associated with diverse cellular activities (m-AAA) metalloprotease complex involved in the maintenance of the mitochondrial proteome [117] and highly expressed in Purkinje cells. The mutation impairs cytochrome c oxidase activity and impairs the cell respiration.

## Dentatorubral Pallidoluysian Atrophy (DRPLA)

DRPLA occurs with highest frequency in the Japanese population (0.2 to 0.7/100,000) [118]. A few cases were reported from European countries [119-125], North America [126] and recently from Turkey [127]. The age of disease onset ranges from 1 to 60 years (mean age is 28.8 years) [119,123]. Patients with earlier onset (below 20 years of age) tend to show myoclonus epilepsy and mental retardation. Patients with late onset (over 40 years of age) tend to present with cerebellar ataxia, choreoathetosis and dementia [128]. Clinical features and the age of onset are significantly correlated with the size of CAG repeats [129]. DRPLA is characterized by prominent anticipation. Head MRI shows atrophy of cerebellum, brainstem, cerebrum and high signal has been shown in periventricular white matter [130]. Unstable expansion of a CAG repeats in the B37 has been demonstrated on chromosome 12p13.31 [118]. And that repeats product an abnormal protein called atrophin 1, which is widely expressed in neurons [131].

## Conclusions

We believe the Harding classification of ataxia syndromes to three major categories, ADCA Type I, ADCA Type II, and ADCA Type III is still helpful to the clinicians taking care of patients presented with SCA phenotypes. In this review we describe the progress in understanding of clinical and pathological phenotypes, and progress in molecular genetic studies related to the ADCA Type I. Further progress in molecular genetic studies will clarify future classifications of these disorders as already indicated in our review (for examples see SCA16 and SCA15). Table 3 lists the known genetic characteristics of the ADCA Type I.

**Table 3:** Genetic Characteristics of ADCA type 1

ADCA	Gene/Gene product	Gene Locus	Repeat Type	Test Availability
SCA1	*SCA1*/ataxin 1	6p23	CAG	Yes
SCA2	*SCA2*/ataxin 2	12q24	CAG	Yes
SCA3	*MJD*/ataxin 3	14q24.3-q31	CAG	Yes
SCA4	*Unkown*	---	---	---
SCA8	SCA8/*ataxin 8*	13q21	CTG	Yes
SCA12	SCA12(PPP2R2B)/*Serine/threonine protein phosphatase 2A, 55 kDA regulatory subunit B, beta isoform*	5q31-q33	CAG	Yes
SCA13	SCA13/*KCNC3 (encodes for a voltage-gated potassium channel)*	19q13.3-q13.4	NA	Yes
SCA16	SCA16/*contactin 4?*	3p26.2-pter	---	---
SCA17	*TBP*/TATA-box binding protein	6q27	CAG	Yes
SCA18	Unknown	7q22-q32	---	---
SCA19	Unknown	1p21-q21	---	---
SCA21	Unknown	7p21.3-p15.1	---	---
SCA23	Unknown	20p13-12.3	---	---
SCA27	*FGF14/*FGF 14	13q34	NA	
SCA28	*SCA28/*unknown	18p11.22-q11.2	---	---

ADCA = autosomal dominant cerebellar ataxia; SCA = spinocerebellar ataxia; PPP2R2B = protein phosphatase 2, regulatory subunit B, beta isoform; KCNC3 = Potassium voltage-gated channel subfamily C member 3; TBP = TATA box binding protein; TATA = thymine adenosine thymine adenosine; FGF14 = fibroblast growth factor 14

For the clinician, a strategy based on Harding's classification of SCA disorders is important to aid in narrowing the diagnostic possibilities. Although genetic testing is the only means of distinguishing in certainty between genotypes, Harding's classification can aid the clinician in developing a genetic molecular testing strategy. Likewise, the vast clinical variability among these syndromes impedes a specific bedside diagnosis; however, knowledge of the clinical characteristics that commonly are associated with each syndrome may streamline the selection of genetic testing. From the standpoint of prevalence, world distribution, and costs a molecular genetic testing for ADCA Type I such as SCA1 - SCA3, SCA8, SCA14, and SCA17 should be considered first. If negative further molecular genetic testing for SCA4, SCA10, SCA12, SCA13, SCA18, and SCA27 can be undertaken. The clinical phenotype may be also used to guide the selection of molecular genetic tests. For example a molecular genetic testing for SCA1, SCA3, SCA 4, SCA8, SCA18 and SCA25 should be considered for an individual with cerebellar ataxia and peripheral neuropathy. Phenotype characterized by a combination of ataxia and epilepsy may indicate need for molecular genetic testing for SCA10, SCA17 and DRPLA. The presence of ataxia and cognitive impairment may suggest the initial selection of molecular genetic studies to SCA1 -, SCA2, SCA13, SCA15/16, SCA17, SCA19, SCA21 and DRPLA. The presence of extrapyramidal sign occurring in SCA2, SCA3, SCA12, SCA15/16, SCA17, SCA21, SCA27, and DRPLA may lead to selection of genetic studies indicative for these disorders. A unique clinical or radiological feature such as action tremor present in SCA12 or dentate calcification seen on head CT in SCA20 may narrow selection of available genetic tests substantially reducing the cost of deriving to diagnosis. Exclusion of treatable and/or structural causes of cerebellar ataxia is mandatory. Neuroimaging studies and routine laboratory testing specifically required to exclude the conditions in the differential diagnosis of cerebellar ataxia should be directed by the history and physical examination.

Unfortunately, no curative therapies have been discovered, though speech and physical therapy, mechanical aids for gait, and symptomatic management of pain and depression can help improve functioning and overall quality of life. Genetic testing is expensive and may not be indicated in many instances where the diagnosis of a neurodegenerative cause is not in question. For affected individuals of child bearing age or where family planning decision making is requested, genetic counseling is essential. Recently, some massively parallel sequencing methods have become available which enable us to screen of thousands of loci for genetic signatures simultaneously. And the methods can reduce the cost and increase the throughput of genomic sequencing [132]. So genetic testing will be less costly and more widely available in the near future.

At this time, the ADCA Type I are no different than most any neurodegenerative syndrome in that the pathophysiology remains uncertain and no curative treatments have been discovered. In the future, more mutations and kindreds with cerebellar ataxia will be discovered and the ADCA type I group will grow, more knowledge of the pathophysiology will mount, and eventually treatments will be forthcoming.

## Competing interests

The authors declare that they have no competing interests.

## Authors' contributions

NRW created the 1^st ^draft of the manuscript that was critically revised by SF. ZKW critically reviewed it.

All authors have read and approved the final manuscript.
